# Assessment of Inter-rater Variability in the Diagnosis of Urinary Tract Infections in the Emergency Department

**DOI:** 10.5811/westjem.53012

**Published:** 2026-04-02

**Authors:** Johnathan M. Sheele, Jesse W. St. Clair, Edward J. Ziegler, Michael M. Mohseni

**Affiliations:** * Mayo Clinic, Department of Emergency Medicine, Jacksonville, Florida; † Mayo Clinic Alix School of Medicine, Jacksonville, Florida

## Abstract

**Introduction:**

Urinary tract infections (UTI) are among the most common bacterial infections diagnosed in the emergency department (ED), yet the urinalysis results can be neither sensitive nor specific for UTI. Our objective was to quantify inter-rater variability of three emergency attending physicians for the clinical diagnosis of UTI, and secondarily to compare the diagnosis made at bedside by the treating clinician with the evaluations of three emergency physician-chart reviewers after the fact.

**Methods:**

Chart reviewers read 18 articles on the diagnosis of UTI before retrospectively evaluating a convenience sample of 473 ED encounters where patients received both a urinalysis and urine culture as part of their ED evaluation. The chart reviewers were blinded to the urine culture results, medications administered and prescribed, and to the treating clinician’s diagnoses. Reviewers were asked to rate the likelihood of UTI based on a 0–4 ordinal scale. A “true positive” UTI occurred when the treating clinician diagnosed the patient with a UTI and the urine culture had ≥10,000 colony-forming units (CFU)/mL of bacteria. We considered a “false positive” to be when the treating clinician diagnosed the patient with a UTI, but the urine culture was < 10,000 CFU/mL of bacteria. A “true negative” occurred when the treating clinician did not diagnose the patient with a UTI, and the urine culture was < 10,000 CFU/mL of bacteria.

**Results:**

Median patient age was 63 years, 355 (75%) were female sex, 409 (86.5%) were White race, and 207 were admitted to the hospital. The inter-rater agreement among the three independent reviewers was high (κ 0.82–0.85) with intraclass coefficient (2,1) = 0.83. However, the reviewers-to-treating clinician agreement was only moderate in the true positives (treating clinician diagnosed patient with a UTI and the patient had a positive urine culture) and lowest in the false positives (treating clinician diagnosed the patient with a UTI, but the urine culture was < 10,000 CFU/mL with κ values of 0.44 and 0.21, respectively). The variables associated with consensus among reviewers were nitrites, leukocyte esterase, and higher urine white blood cells.

**Conclusion:**

There was high consensus among reviewers about the likelihood of a urinary tract infection, but lower consensus when comparing reviewers’ impressions with those of the treating clinician. At bedside emergency clinicians were more likely to diagnose a UTI with a resultant negative urine culture. Further research is needed to improve the diagnostic accuracy of UTI in the emergency department.

## INTRODUCTION

Urinary tract infections (UTI) are among the most common problems evaluated in the emergency department (ED) and result in high empiric antibiotic use.^1^–^3^ Yet diagnosing UTI accurately is difficult: Symptoms often overlap with other conditions, presentations are atypical in older or catheterized patients, and culture results arrive after key decisions have been made.^4^–^7^ These realities create fertile ground for disagreement among clinicians about who truly has an infection vs colonization, or whether there is an alternative diagnosis. (eg, in an ED cohort in the United Kingdom, 60–70% of patients treated for UTI ultimately lacked microbiological or clinical confirmation^8^). Within this context sexually transmitted infections may be under-recognized.^9^ Inter-rater reliability (IRR)—the extent to which different clinicians reach the same conclusion on the same patient—is a practical barometer of diagnostic quality in this setting.^10^–^11^

The consequences of unreliability are substantial. Overdiagnosis exposes patients to unnecessary antibiotics, with attendant risks of adverse drug events and antimicrobial resistance, whereas under-recognition of true infections can delay appropriate therapy and worsen outcomes.^12^–^14^ In the high-throughput emergency setting, where decisions must be made quickly and often with incomplete data, improving diagnostic reliability supports both safer individual care and better stewardship.

Prior work shows that agreement on UTI is far from perfect. Studies comparing frontline ED assessments with expert review demonstrate only moderate concordance, even when experts themselves show high internal agreement.^11^ Diagnostic conclusions also change across care settings: Among admissions for suspected infection, the presumed source is frequently reclassified by discharge, with urinary sources commonly reassigned once full work-ups are available.^15^ At the bedside, agreement among clinicians is mixed, with objective signs such as fever being rated consistently reliable, while subjective findings of suprapubic discomfort or flank tenderness show only fair concordance.^16^ This highlights the variability inherent in clinical examination.

Common diagnostic aids and criteria only partially solve the problem. Urinalysis elements and dipsticks, although widely available, have limited specificity and are confounded by asymptomatic bacteriuria, contamination, and comorbid conditions.^17^–^18^ Routine urine testing itself can be associated with inappropriate antibiotic use and longer ED length of stay.^19^ Consensus definitions designed for surveillance or long-term care settings (including the Loeb minimum criteria) and checklists perform poorly when translated to ED decision-making in older adults, while guideline recommendations (including NICE NG109) caution against routine dipstick-driven diagnosis in several adult groups.^11^,^20^–^21^ Conversely, structured tools that standardize how data are weighed can improve concordance, illustrating that how we define and apply criteria materially influences inter-rater reliability.^22^ Professional societies including the Infectious Diseases Society of America (IDSA) and the European Association of Urology (EAU) have called for standardized approaches to defining and diagnosing UTIs, particularly in high-volume settings such as the ED.^18^,^23^

**Population Health Research Capsule**

What do we already know about this issue?

*Urinary tract infections (UTI) are frequently misdiagnosed in the emergency department (ED)*.

What was the research question?

*We sought to quantify inter-rater variability for UTI diagnosis between the treating clinician and three emergency physician chart reviewed*.

What was the major finding of the study?

*The agreement among three reviewers reporting the likelihood of UTI was κ 0.82–0.85 (quadratic-weighted)*.

How does this improve population health?

*Accurate diagnosis of UTI can reduce inappropriate antibiotic use*.

Despite this recognition that reliability matters, few studies have investigated the inter-rater reliability among emergency clinicians in the diagnosis of UTI and described how their judgment aligns with that of the treating clinician at the point of care. The objective of our study was to quantify inter-rater reliability for UTI diagnosis in an ED cohort by measuring agreement among three independent emergency physicians and comparing these assessments to the treating clinician. We further sought to identify which patient and laboratory factors, including urinalysis elements, may be associated with greater consensus or discordance. By focusing on reliability as a clinical quality signal, we aimed to highlight practical targets for improving diagnostic accuracy and antibiotic stewardship in emergency care.

## METHODS

The study was approved by the Mayo Clinic Institutional Review Board. We followed the 2015 Standards for Reporting of Diagnostic Accuracy and emergency medicine retrospective research guidelines.^24^ Urine samples were collected from Mayo Clinic EDs in Rochester, MN. All patient encounters took place between December 2024–May 2025. A convenience sample of ED patients who had a urinalysis and urine culture performed as part of their ED evaluation had their ED chart retrospectively reviewed with the following clinical information withheld from review: the urine culture and sensitivity results; the emergency clinician’s diagnoses; and all medications and procedures administered during the ED encounter or provided at discharge. The reviewers had access to the past medical history, past surgical history, the reason for the ED encounter provided in triage, the patient’s history, review of systems, physical exam, patient demographics, vital signs, laboratory results, and the radiology reports for computed tomography and ultrasound studies, and they were aware of the study objectives. The clinical data was reviewed by three board-certified attending physicians in emergency medicine who reviewed the following articles on the diagnosis and management of UTIs and asymptomatic bacteriuria (supplement 1). The chart reviewers were not blinded to the study hypothesis.

The three chart reviewers were asked to provide a 0–4 score for each chart using the following instructions:

There is no universal definition of a UTI. Based on your review of the literature and clinical experience do you think the patient most likely has (4 = definite uncomplicated or complicated UTI; 3 = likely uncomplicated or complicated UTI; 2 = may have/unsure uncomplicated or complicated UTI; 1 = asymptomatic bacteriuria (no UTI but will have ≥10,000 CFU/mL bacteriuria on urine culture), 0 = no UTI and urine culture with < 10,000 CFU/mL bacterial growth.

Both the ordinal 0–4 scale was used as well as a binary scale (yes [3–4] and no [0–2]). Patients were considered to have been diagnosed with a UTI (yes vs no) if they received a diagnosis of UTI, pyelonephritis, ureteritis, pyonephrosis, renal abscess, or cystitis. Positive urine cultures were when the urine culture grew ≥ 10,000 colony-forming units (CFU)/mL of bacteria, and < 10,000 CFU/mL were considered negative. If the urine white blood cells (WBCs) and red blood cells (RBCs) results were reported as a range, we chose to use the mean value of the range. If > 100 cells were reported, they were reclassified as 100 cells. These changes were allowed so that they could be modeled as continuous variables. We considered a “true positive” to be when the treating clinician diagnosed the patient with a UTI and the urine culture was positive. We considered a “false positive” to be when the clinician diagnosed the patient with a UTI, but the urine culture was negative. We considered a “true negative” to be when the clinician did not diagnose the patient with a UTI, and their urine culture was negative (ie, no UTI present). The primary outcome was assessing IRR between the independent reviewers, and our secondary outcome was comparing the results of the independent emergency-physician reviewers with the treating clinician in the ED.

### Statistical analysis

Continuous and ordinal variables are summarized as median (IQR). Categorical variables are reported as n (%). Inter-rater reliability for the 0–4 reviewer ratings was assessed pairwise with quadratic-weighted Cohen κ; 95% confidence intervals were obtained via nonparametric bootstrap (800 resamples). We also estimated Intraclass correlation coefficient (ICC) (2,1) (two-way random, absolute agreement) across all three reviewers, with bootstrap CIs. The Bowker test of symmetry evaluated marginal homogeneity. After dichotomization to UTI/no UTI, we quantified pairwise agreement with Cohen’s κ, its asymptotic standard error (SE), Wald 95% CIs, and McNemar χ^2^. For three and four raters we used Fleiss κ with approximate SE, Wald CIs, and corresponding *P* values. To explore factors associated with subject-level consensus across the clinician and three reviewers, we computed per-subject agreement (Pi) as the sum of squared category proportions and modeled high agreement (Pi ≥ 0.75) with multivariable logistic regression, including age, sex, race, ED disposition, and urinalysis elements (excluding urine protein and culture result). Results are reported as odds ratios (OR) with 95% CIs. All tests were two-sided with α = 0.05. Missing data were excluded listwise for each analysis without imputation.

## RESULTS

The median age was 63 years (interquartile range IQR 42–78). Of those patients included in the study, 355 (75%) were female sex, and 409 (86.5%) were White; 207 were admitted into the hospital. There were 145 (30.7%) encounters where the treating clinician diagnosed a patient with a UTI, of whom 132 had ≥ 10,000 CFU/mL bacteriuria on urine culture. In 121 (25.6%) encounters the clinician did not diagnose a UTI and the patient had ≥10,000 CFU/mL of bacteriuria on urine culture. There were 253 (53.5%) encounters with ≥ 10,000 CFU/mL bacteriuria on urine culture. Results are summarized in Table 1. A positive urine culture was noted among those diagnosed with a UTI in 135/145 (93.1%) among treating clinicians; 92/96 (95.8%) for Reviewer 1; 119/121 (98.3%) for Reviewer 2; and 92/97 (94.8%) for Reviewer 3.

The results of the three blinded reviewers on the likelihood of UTI are listed in Table 2. Most patients were categorized as 0 (no UTI) or 3–4 (likely/definite UTI). Table 3 shows there was high agreement between the blinded reviewers when the ordinal 0–4 categorization was used with the quadratic-weighted Cohen κ (0.82–0.85) and > 94% observed agreement. The ICC (2,1) was 0.83 when all three reviewers were compared.

We compared the independent reviewers’ binary responses for UTI (present = 3–4, not present = 0–2) and the treating ED clinician diagnosis (UTI present or not present) (Table 4). Agreement between the clinicians was moderate with Cohen κ values (0.60–0.81). The treating clinician more frequently diagnosed UTI than the independent reviewers (145 vs 96, 121, and 97; respectively). Fleiss κ with all four reviewers only showed moderate reliability (.69).

There was only moderate agreement among the independent reviewers, Fleiss κ of 0.44, for those diagnosed with a true positive UTI (Table 5). There was high agreement among the reviewers for those diagnosed with a true negative (ie, no UTI) with a Fleiss κ of 1.0. There was low agreement among reviewers for those diagnosed with a false positive UTI (diagnosed with a UTI but a negative urine culture), with a Fleiss κ of 0.21.

Table 6 shows the variables associated with concordance between the treating clinician and the three reviewers for UTI. Higher age, nitrite positive, leukocyte esterase, urine WBCs, and urine bacteria present were significantly associated with reviewer concordance in the model (*P* < .05) but all were associated with reduced agreement (odds ratioOR < 1). Other variables, including sex, disposition status, clarity, hemoglobin, glucose, ketone, and urine RBCs, were not significantly associated.

## DISCUSSION

Blinded emergency physician reviewers had higher agreement when assessing the likelihood of UTI when using an ordinal 0–4 scale, but agreement dropped when the results were dichotomized to UTI vs no UTI in a convenience sample of ED patients. These findings are consistent with prior studies demonstrating diagnostic uncertainty and discordance in diagnosing UTIs in acute care settings among clinicians.^3^–^4^,^6^,^11^,^18^–^19^ Agreement among reviewers was highest in patients with urine culture-confirmed UTIs and no UTIs and negative urine cultures, and was lowest among those patients who were diagnosed with a UTI but had negative urine cultures. These findings are consistent with prior research showing that dipstick-driven or symptom-driven diagnostics can over-diagnose UTIs, especially among older adults and patients with nonspecific symptoms.^3^,^19^–^21^ The regression analysis found that those variables most associated with a UTI (eg, nitrites, urine WBCs, and leukocyte esterase) were most likely to be associated with agreement among the independent reviewers.^25^,^26^

While the treating clinicians at bedside in the ED diagnosed more UTIs (n = 145) than any of the independent reviewers (range: 96–121), the percentages of encounters where a UTI was diagnosed (with positive urine culture noted) was high among both groups: 93.1% for the treating clinicians and 94.8–98.3% for the reviewers. However, there was moderate agreement between independent chart reviewers and the treating clinician on whether the patient had a UTI [κ = 0.64 (0.56–0.72)]. One potential explanation for this observation is that treating clinicians are at the bedside, treating the patient in real time, while the reviewers were looking at data retrospectively. Bedside clinicians are subject to external pressures, such as having to make decisions quickly and to accommodate patients’ expectations, while these factors are not present for reviewers. Our study is in line with previous research on inter-rater reliability.^10^,^27^

There are no universal diagnostic criteria for a UTI. Multiple professional societies and government agencies have developed slightly differing guidelines, although not all apply to the ED. The latest update on UTI from the Infectious Disease Society of America (IDSA) received input from the Society of Academic Emergency Medicine (SAEM).^28^ Because the SAEM UTI diagnostic guidelines were published in 2013, they are likely outdated; emergency physicians should reference the updated IDSA guidelines when considering a diagnosis of UTI.^25^

The updated IDSA uncomplicated UTI criteria include local bladder signs and symptoms such as urgency, dysuria, frequency, and abdominal pain without fever from the UTI in men and women without catheters, nephrostomy tubes, and stents.^28^ There should be no signs or symptoms of systemic illness, costovertebral angle tenderness, or flank pain.^28^ The diagnosis does not require specific findings on urinalysis or a positive urine culture.^28^ Complicated UTIs have symptoms suggesting extension beyond the bladder including fever, chills, rigors, hemodynamic instability, flank pain, costovertebral angle tenderness, pyelonephritis, urinary catheters, neurogenic bladder, urinary obstruction or retention.^28^ These guidelines are similar to the European Urological Association guidelines on urological infections with the exception that a positive urine culture is used to confirm infection, even though the CFU/mL bacteriuria cutoff counts vary by sex, method of urine of collection, and organism growing. Symptom-driven diagnosis of UTI in the ED likely overtreats patients who have an alternative diagnosis causing genitourinary symptoms such as sexually transmitted infections. The accuracy of the latest IDSA UTI guidelines needs to be further studied in the ED.

## LIMITATIONS

Treating clinicians in the ED diagnosed patients with a UTI or not (a binary decision), whereas the emergency-physician chart reviewers used a 0–4 scale, which may limit appropriate comparisons. Our study involved a single system and was retrospective. We analyzed a convenience sample of ED patients who had a urinalysis and urine culture as part of the ED evaluation; so our results may have limited generalizability. Chart reviewers were not blinded to the study hypothesis, but reviewers were blinded to culture and treatment, and spectrum and incorporation biases are possible. Our patients were typically older and were predominantly White, which also may affect results. All our independent reviewers were board-certified emergency attending physicians, whereas some of the treating clinicians could have been advanced practice clinicians, which could have influenced the results. Finally, we conducted our research before the latest IDSA guidelines were published; these guidelines do not necessitate the presence of a positive urine culture as was used in our study.

## CONCLUSION

Blinded emergency physician chart reviewers showed high agreement on likelihood of urinary tract infection, but agreement with treating clinicians was only moderate and lowest in false-positive strata. Variables highly associated with UTI (nitrites, leukocyte esterase, urine bacteria, and urine WBCs) were all statistically significant in our multivariate model but were associated with lower inter-rater consensus for UTI.

## Supplementary Information



## Figures and Tables

**Table 1:** Summary of patients examined (n = 473) in an emergency department study comparing the diagnosis made by the treating clinician at bedside and later assessment by three chart reviewers.

Variable	Category	Median (IQR) or n (%)
Age (years)		63.0 (42.0–78.0), 473
Sex	Female (vs. male)	355 (75.1%)
Race binary	White (vs. non-white)	409 (86.5%)
ED disposition	Discharged or AMA (vs. admit or observation)	264 (56.1%)
Urine clarity	Clear (vs. cloudy)	385 (83.1%)
Hemoglobin (0–4+)		0.0 (0.0–2.0), 468
Glucose (0–1000 mg/dL)		0.0 (0.0–0.0), 472
Ketone (0–160 mg/dL)		0.0 (0.0–5.0), 472
Urine pH (4.6–9)		5.8 (5.4–6.4), 473
Nitrites	Negative (vs. positive)	398 (85.0%)
Leukocyte esterase (0–4+)	0	0 (0–3), 469
Urine RBCs (0–100 cells/HPF)		3.0 (0.0–6.0), 471
Urine WBCs (0–100 cells/HPF)		2.0 (0.0–15.0), 470
Urine Bacteria	Absent (vs. present)	303 (64.1%)
max CFU/mL	Positive (vs. negative)	253 (53.5%)

*AMA*, against medical advice*; HPF*, high powered field; *IQR*, interquartile range; *RBC*, red blood cell; *WBC*, white blood cell.

**Table 2:** Results of blinded reviewer assessment of the likelihood of urinary tract infection being present on chart review.

Rater	no UTI and urine culture with < 10,000 CFU/mL bacterial growth (0)	asymptomatic bacteriuria (no UTI but will have ≥ 10,000 CFU/mL bacteriuria on urine culture (1)	may have/unsure uncomplicated or complicated UTI (2)	likely uncomplicated or complicated UTI (3)	definite uncomplicated or complicated UTI (4)
Reviewer 1	299 (63.21%)	64 (13.53%)	14 (2.96%)	20 (4.23%)	76 (16.07%)
Reviewer 2	312 (65.96%)	11 (2.33%)	29 (6.13%)	22 (4.65%)	99 (20.93%)
Reviewer 3	254 (53.70%)	66 (13.95%)	56 (11.84%)	39 (8.25%)	58 (12.26%)
Total	865 (60.96%)	141 (9.94%)	99 (6.98%)	81 (5.71%)	233 (16.42%)

*CFU*, colony-forming unit; *UTI*, urinary tract infection.

**Table 3:** The inter-rater reliability between reviewers for the likelihood of urinary tract infection for emergency department patients using an ordinal 0–4 scale.

Comparison	Statistic used	κ (95% CI)	SE	*P* value	Observed agreement (Po)	Expected agreement (Pe)	Bowker symmetry χ^2^ (*P*)
1 vs 2	Quadratic-weighted Cohen κ (ordinal)	0.82 (0.77–0.86)	0.02	<.001	0.94	0.68	60.78 (.00)
1 vs 3	Quadratic-weighted Cohen κ (ordinal)	0.85 (0.81–0.88)	0.02	<.001	0.96	0.73	61.68 (.00)
2 vs 3	Quadratic-weighted Cohen κ (ordinal)	0.83 (0.79–0.87)	0.02	<.001	0.95	0.70	96.95 (.00)
1, 2, 3	ICC(2,1) two-way random, absolute agreement (ordinal treated continuous)	ICC 0.83 (0.79–0.86)	0.02	<.001	NA	NA	NA

*ICC*, intraclass correlation coefficient.

**Table 4:** The inter-rater reliability of the independent reviewers and the treating clinician in the emergency department when the reviewers’ responses were binarized as urinary tract infection (UTI)/no UTI. Cohen κ used unless other reported.

Comparison	κ (95% CI)	SE	*P* value	Observed agreement (Po)	Expected agreement (Pe)	McNemar χ^2^ (*P*)
1 vs 2	0.70 (0.62–0.78)	0.04	<.001	0.89	0.65	11.29 (< .001)
1 vs treating clinician	0.60 (0.51–0.68)	0.04	<.001	0.85	0.61	31.56 (< .001)
1 vs 3	0.81 (0.74–0.88)	0.03	<.001	0.94	0.68	.00 (1.0)
2 vs treating clinician	0.74 (0.67–0.81)	0.03	<.001	0.89	0.59	10.58 (< .001)
2 vs 3	0.67 (0.59–0.75)	0.04	<.001	0.88	0.64	9.45 (< .001)
3 vs treating clinician	0.64 (0.56–0.72)	0.04	<.001	0.86	0.61	33.47 (< .001)
1, 2, 3*	0.72 (0.63–0.81)	0.05	<.001	0.90	0.66	NA
1, 2, 3, ED treating clinician*	0.69 (0.63–0.75)	0.03	<.001	0.89	0.63	NA

* Fleiss κ (multi-rater, binary).

*SE*, standard error.

**Table 5:** The inter-rater reliability of all three reviewers for urinary tract infection (UTI) (likely or definitive UTI) vs. unlikely or no UTI using Fleiss κ (multi-rater, binary) when comparing true positive, true negative (no UTI), and false positive diagnoses by the treating clinician in the emergency department.

	κ (95% CI)	SE	*P* value	Observed agreement (Po)	Expected agreement (Pe)
True positive (treating clinician diagnosed UTI and urine culture ≥ 10,000 CFU/mL) (n = 132)	0.44 (0.29–0.59)	0.08	<.001	0.76	0.57
True negative (no UTI) (treating clinician did not diagnose UTI and urine culture < 10,000 CFU/mL) (n = 207)	1.00 (0.20–1.80)	0.41	0.01	1.00	0.99
False positive (treating clinician diagnosed UTI and urine culture < 10,000 CFU/mL) (n = 13)	0.21 (−0.34–0.76)	0.28	0.45	0.74	0.67

*CFU*, colony-forming units*; SE*, standard error, *UTI*, urinary tract infection.

**Table 6:** Variables associated with high inter-rater agreement between three reviewers and the treating clinician in the emergency department for urinary tract infection using logistic regression.

Variable	OR (95% CI)	*P* value
Age (years)	0.98 (0.97–1.00)	.01
Sex (male vs. female)	1.25 (0.66–2.34)	.49
Race binary (White vs. non-White)	2.63 (0.92–7.50)	.07
ED disposition status (admitted/observation vs discharge/AMA)	0.78 (0.46–1.32)	.36
Urine clarity (clear vs. cloudy)	1.15 (0.58–2.27)	.69
Hemoglobin	0.76 (0.31–1.82)	.53
Glucose	1.00 (1.00–1.00)	.43
Ketone	1.01 (0.99–1.02)	.33
Nitrites (positive vs. negative)	0.27 (0.15–0.49)	< .001
Leukocyte esterase	0.27 (0.09–0.80)	.02
Urine RBCs	Unstable estimate*	.84
Urine WBCs	0.99 (0.98–0.99)	< .001
Urine bacteria (present vs. absent)	0.39 (0.23–0.67)	< .001

* Urine RBCs odds ratio unstable due to sparse data and quasi-complete separation.

*AMA*, against medical advice; *RBC*, red blood cell; *WBC*, white blood cell.
