# Supplementary material for: Assessment of Inter-rater Variability in the Diagnosis of Urinary Tract Infections in the Emergency Department
Source: West J Emerg Med. 2026 Apr 2;27(3):676–83. doi: 10.5811/westjem.53012 (PMC13246192; doi:10.5811/westjem.53012)
Supplement: Supplementary file 1 [file wjem-27-676-s001.docx]

Supplement 1. List of resource articles on the diagnosis and management of urinary tract infections and asymptomatic bacteriuria.

1. Cheng B, Zaman M, Cox W. Correlation of Pyuria and Bacteriuria in Acute Care. *Am J Med*. Sep 2022;135(9):e353-e358. doi:10.1016/j.amjmed.2022.04.022

2. Kranz J, Bartoletti R, Bruyere F, et al. European Association of Urology Guidelines on Urological Infections: Summary of the 2024 Guidelines. *Eur Urol*. Jul 2024;86(1):27-41. doi:10.1016/j.eururo.2024.03.035

3. Schulz L, Hoffman RJ, Pothof J, Fox B. Top Ten Myths Regarding the Diagnosis and Treatment of Urinary Tract Infections. *J Emerg Med*. Jul 2016;51(1):25-30. doi:10.1016/j.jemermed.2016.02.009

4. Giesen LG CG, Dimitrov BD, van de Laar FA, Fahey T. . Predicting acute uncomplicated urinary tract infection in women: a systematic review of the diagnostic accuracy of symptoms and signs. *BMC Family Practice*. 2010;11(1):78.

5. Redwood R CK. The Diagnosis and Treatment of Adult Urinary Tract Infections in the Emergency Department. *Emergency Medicine Clinics of North America*. 2024;42(2):209-30.

6. Horan TC, Andrus M, Dudeck MA. CDC/NHSN surveillance definition of health care-associated infection and criteria for specific types of infections in the acute care setting. *Am J Infect Control*. Jun 2008;36(5):309-32. doi:10.1016/j.ajic.2008.03.002

7. Patel R, Polage CR, Dien Bard J, et al. Envisioning Future Urinary Tract Infection Diagnostics. *Clin Infect Dis*. Apr 9 2022;74(7):1284-1292. doi:10.1093/cid/ciab749

8. Nicolle LE, Gupta K, Bradley SF, et al. Clinical Practice Guideline for the Management of Asymptomatic Bacteriuria: 2019 Update by the Infectious Diseases Society of America. *Clin Infect Dis*. May 2 2019;68(10):1611-1615. doi:10.1093/cid/ciz021

9. Werneburg GT, Wagenlehner F, Clemens JQ, Harding C, Drake MJ. Towards a Reference Standard Definition of Urinary Tract Infection for Research. *Eur Urol Focus*. Jan 2025;11(1):71-75. doi:10.1016/j.euf.2024.09.010

10. Bilsen MP, Jongeneel RMH, Schneeberger C, et al. Definitions of Urinary Tract Infection in Current Research: A Systematic Review. *Open Forum Infect Dis*. Jul 2023;10(7):ofad332. doi:10.1093/ofid/ofad332

11. Holm A, Siersma V, Cordoba GC. Diagnosis of urinary tract infection based on symptoms: how are likelihood ratios affected by age? a diagnostic accuracy study. *BMJ Open*. Jan 8 2021;11(1):e039871. doi:10.1136/bmjopen-2020-039871

12. Blok B C-DD, Del Popolo G, Groen J, Hamid R, Karsenty G, Kessler TM, Pannek J. EAU Guidelines Neuro-Urology. *European Association of Urology*. 2015:1-60.

13. G. Bonkat RB, F. Bruyère, T. Cai,, S.E. Geerlings BK, J. Kranz, S. Schubert,, A. Pilatz RV, F. Wagenlehner, Guidelines Associates: K. Bausch WD, J. Horváth LL, G. Mantica, T. Mezei. EAU Guidelines on Urological Infections. 2023;

14. Alidjanov JF NK, Pilatz A, Radzhabov A, Zamuddinov M, Magyar A, Tenke P, Wagenlehner FM. . Evaluation of the draft guidelines proposed by EMA and FDA for the clinical diagnosis of acute uncomplicated cystitis in women. *World Journal of Urology*. 2020;38(1):63-72.

15. Uncomplicated Urinary Tract Infections: Developing Drugs for Treatment Guidance for Industry (2019).

16. Bilsen MP LM, Conroy S. Guideline commentary on updated NICE guidelines for urinary tract infections. *Age and Ageing*. 2023;52(3):1-3.

17. Urinary tract infections in adults 1-27 (2024).

18. Advani SD, North R, Turner NA, et al. Performance of Urinalysis Parameters in Predicting Urinary Tract Infection: Does One Size Fit all? *Clin Infect Dis*. Apr 26 2024;doi:10.1093/cid/ciae230
